# Halitosis in Children Undergoing Full Mouth Rehabilitation under General Anesthesia

**DOI:** 10.3390/children8020149

**Published:** 2021-02-17

**Authors:** Noura A. AlMadhi, Ayman M. Sulimany, Hamad A. Alzoman, Omar A. Bawazir

**Affiliations:** 1 Department of Pediatric Dentistry and Orthodontics, College of Dentistry, King Saud University, Riyadh 11545, Saudi Arabia; asulimany@ksu.edu.sa (A.M.S.); obawazir@ksu.edu.sa (O.A.B.); 2 Department of Periodontics and Community Dentistry, College of Dentistry, King Saud University, Riyadh 11545, Saudi Arabia; HAlzoman@ksu.edu.sa

**Keywords:** children, full mouth rehabilitation, general anesthesia, halitosis, perception

## Abstract

Interventions and management modalities of pediatric halitosis have been suggested in the literature, however, the effect of full mouth rehabilitation (FMR) under general anesthesia (GA) on pediatric halitosis was not reported. Therefore, the present study was conducted to investigate parents’ perceptions of their child’s halitosis before and after FMR under GA; and to evaluate the effect of FMR on clinical halitosis. Fifty-seven children between 3–8 years old, scheduled for FMR under GA, were included after satisfying the inclusion criteria and upon parental consent. Parents’ perception of halitosis in their children was evaluated using a standardized questionnaire and a breath sample was collected to assess the level of volatile sulfur compounds (VSCs) using OralChroma^TM^ before and after FMR under GA. Sixty percent (*n* = 34) of the parents perceived halitosis in their children before FMR and about 80% (*n* = 27) of them reported improvement in halitosis after FMR. Clinical halitosis was detected in 84.2% (*n* = 48) of the sample before treatment. A statistically significant reduction in halitosis was found in 56.3% (*n* = 27) of the children after treatment (*p* < 0.001). In conclusion, majority of parents perceived an absence or reduction of halitosis in their children following FMR and significant improvement of clinical halitosis.

## 1. Introduction

Halitosis is a condition characterized by foul-smelling or offensive odors emanating from the mouth or breath. According to the literature, halitosis can affect all age groups, including children [1]. The prevalence of halitosis among pediatric populations has been investigated in different regions of the world with varying estimations [2,3,4,5,6,7]. In Japan, the prevalence of halitosis in children ranged between 8% and 45% [4,5,6], while it was reported as 41% In India [7]. The prevalence of halitosis among pediatrics in Italy was 38% [3], while it was reported as approximately 15% in Turkish children [2]. The discrepancy between the prevalence of halitosis in children could be due to the lack of standardization in halitosis detection and threshold criteria.

The most commonly used methods to evaluate halitosis in children are organoleptic assessment [2,4,5,6,7] and sulfide monitoring devices [3,5,8]. The organoleptic method depends on odor judges who smell the air exhaled from the mouth and nose, while the sulfide monitor is a device that measures the total volatile sulfur compounds (VSCs). Halitosis is known to be associated with high levels of VSCs, with hydrogen sulfide (H_2_S), methyl mercaptan (CH_3_SH) and dimethyl sulfide (CH_3_SCH_3_) as the major VSCs causing halitosis [1,9]. Gas chromatography is considered the gold standard method for differentiating between the three VSC gases associated with halitosis [10]. OralChroma^TM^ is a commercially available portable gas chromatography that can be used in the clinic to detect and measure the major VSCs, which consequently aids in the monitoring and management of halitosis [11,12,13]. 

Interventions and management modalities for halitosis have been suggested in the literature. Since oral halitosis is related to the degradation of components of epithelial cells, salivary or serum proteins and food debris by wide range of oral anaerobic microorganisms to produce VSCs, management modalities varied between mechanical and chemical reduction of bacteria and its substrate to use products that mask or neutralize VSCs [14,15].

In general, few studies have assessed different interventions or management methods to reduce halitosis in the pediatric population compared with adults. Some studies have found that improved oral hygiene measures, tongue cleaning and non-surgical periodontal treatment show significant reductions in halitosis [8,16,17,18]. Other study have failed to show improvements in halitosis following tongue debridement [19]. 

According to the literature, the positive impact of full mouth rehabilitation (FMR) under general anesthesia (GA) on oral health-related quality of life (OHRQoL) and the general health of children and their families has been reported [20,21,22,23,24]. Additionally, systematic reviews have reported similar conclusions, wherein the improvement of children’s oral health resulted in an improvement of physical, emotional and social quality of life [21,22]. Only one study evaluated oral bad breath subjectively as an item in their quality of life questionnaires and they found an improvement in the malodor [24]. However, no study has evaluated the parents’ perception of halitosis in their children subjectively nor evaluated the clinical halitosis objectively before and after FMR under GA. Therefore, the present study aimed to: (1) investigate parents’ perceptions of their child’s halitosis before and after FMR under GA and (2) evaluate the effect of FMR on clinical halitosis in children.

## 2. Materials and Methods

### 2.1. Study Design 

Fifty-seven children who were scheduled for FMR under GA due to their uncooperative behavior in the dental clinic or extensive dental needs in the Dental University Hospital (DUH) at King Saud University (KSU), Riyadh, Saudi Arabia were included in this study after fulfilling the following inclusion criteria: healthy children, three to eight years old and their parents agreed to participate in the study and were cooperative during the clinical examination and halitosis measurements. Children with a history of tonsillitis, upper respiratory infection, allergic rhinitis or gastrointestinal disorders, Candida infections or antibiotic use one month before the examination were excluded. Parents were contacted by phone one day before the GA screening appointments and initial verbal approval was obtained. Risks and benefits were explained to the parents and halitosis test instructions were given (not to eat, drink, brush teeth or rinse their mouths on the appointment day). Clinical examination and breath sample were performed early morning in the GA screening session (one day prior to the GA) and a written consent form was signed by the parents. Ethical approval was obtained from the Institutional Review Board (IRB. No. E-18-3456) and the research was registered in the College of Dentistry Research Center in KSU (CDRC. No. PR 0085). 

### 2.2. Clinical Examinations and Breath Sample Collection

A self-administered questionnaire was carried out first to obtain demographic data and parents’ perceptions about halitosis in their children. Then, children were examined in the dental chair by the primary investigator (NA) to collect the following data: dental caries score using dmft/DMFT (decayed, missing, filled teeth) Index by the WHO (2013) [25], oral hygiene level using the Simplified Debris Index (DI-S) by Greene and Vermillion (1964) [26], gingival health condition using the Modified Gingival Index (MGI) by Lobene et al. (1986) [27] and tongue coating using the Winkel Tongue Coating Index (WTCI) by Winkel et al. (2003) [28]. The intra-examiner reliability of the clinical parameters was assessed by the correlation coefficient and scored an average measure of 0.9, indicating excellent reliability. Then, parents and children were escorted to the halitosis clinic. An illustrative video was shown on how the breath sample would be obtained and measured using an OralChroma^TM^ device and the child was then asked to close his/her mouth for 1 min. A breath sample was then obtained using a disposable 1 mL plastic syringe and then injected immediately into the inlet of the OralChroma^TM^ device (CHM-2, Nissha FIS, Inc. Abimedical Corporation, Osaka, Japan). VSCs were analyzed and concentration values of H_2_S, CH_3_SH and CH_3_SCH_3_ were recorded in parts per billion (ppb). Halitosis was considered present clinically if one or more gas was equal to or higher than the following cognitive threshold according to the manufacturer’s instructions: H_2_S ≥ 112 ppb, CH_3_SH ≥ 26 ppb and CH_3_SCH_3_ ≥ 8 ppb. Oral hygiene instructions were provided to all participating children and their parents.

### 2.3. Follow-up Visit

The follow-up visit was 4–6 weeks after the FMR procedure. Parents were contacted one day before their follow-up appointment and similar pre-halitosis assessment instructions were given. During this visit (early morning), the same parent who filled out the pre-operative questionnaire was asked to fill in the post-operative questionnaire to evaluate their perception of halitosis after FMR. Then, a clinical examination was performed and a breath sample was taken by the same investigator to record changes in the clinical parameters and changes in the levels of VSCs. The participating children were rewarded at the end of the appointment with stickers and balloons as a thankful gesture for their cooperation. 

### 2.4. Sample Size and Statistical Analysis

Differences in the means between two dependent variables (matched pairs) were compared at α = 0.05 with a 0.4 effect size and power of 0.9 [29]. The sample size was calculated to be 50 children in order to detect significance. Due to an anticipated 20% loss to follow-up, the sample size was increased to 60 children. For all statistical analyses, the SPSS (version 22.0, IBM Inc., Chicago, IL, USA) program was used. Normality of the data was checked using the Kolmogorov–Smirnov test. Frequencies and percentages of categorical variables with means and standard deviations or medians and interquartile ranges (IQRs) of continuous variables were used as descriptive statistics. The modified gingival index was dichotomized into 0 = no gingival inflammation and 1 = inflamed gingiva for the purpose of the analysis. The data were skewed; therefore, non-parametric tests were used to observe significant differences. The McNemar test was used for categorical data and the Wilcoxon signed ranked test was used for continuous data. A *p*-value < 0.05 was considered statistically significant. 

## 3. Results

### 3.1. Demographic Data

Fifty-seven Saudi children between 3 and 8 years of age were included in this study (mean age 5.4 ± 1.6 years), wherein 68.4% (*n* = 39) were females and 31.6% (*n* = 18) males (Table 1). The questionnaires were mostly filled out by fathers 72% (*n* = 41) and the rest were filled out by mothers 28% (*n* = 16). 

### 3.2. Parents’ Perception of Halitosis in Their Children

Sixty percent of parents (*n* = 34) perceived halitosis in their children before FMR (Table 1). Approximately 80% (*n* = 27) of them reported improvement in perceived halitosis after FMR. None of the parents (*n* = 23) who failed to perceive halitosis before rehabilitation reported any deteriorations in oral breath after the treatment. 

### 3.3. Breath Sample Analysis

Halitosis was initially detected in 84.2% (*n* = 48) of children prior to FMR under GA by measuring the level of VSCs using OralChroma^TM^. Following the FMR procedure, statistically significant reductions in clinical halitosis were found in 56.3% (*n* = 27) of children (*p* < 0.001) (Table 2). When VSCs were measured as continuous variables, a statistically significant reduction in the level of VSCs for all gases was found (*p* < 0.001) (Table 3).

### 3.4. Clinical Parameters 

The clinical parameters before and after FMR are presented in Table 4. The oral hygiene was fair or poor for all participants before treatment; however, a statistically significant improvement was noted in half of the participants following treatment (*n* = 29, 50.9%). MGI and the Winkel Tongue Coating Index were significantly reduced post-FMR (*p* < 0.001). 

## 4. Discussion

Halitosis in children is a common condition, although it does not receive the same attention in the literature as adults. This study may be the first to investigate halitosis subjectively by evaluating parents’ perception and objectively by measuring the VSCs concentrations using the OralChorma^TM^ before and after FMR under GA.

In the present study, parents perceived halitosis in 60% of their children before providing treatment. This finding is in agreement with that reported by Lin et al., who found that 61% of mothers complained of bad breath in their children [19]. Contradicting this, Aliyu and Lawal (2018) reported that only twelve percent of Nigerian parents perceived halitosis in their children [30]. The variations in the results could be attributed to differences in the emotional status and olfactory sensation of the evaluators. Furthermore, the culture of the studied populations and the time of the evaluation play significant roles in halitosis perception. In addition, the oral health status of the children was not indicated. 

Clinical halitosis was detected by OralChroma^TM^ in the majority of children before FMR under GA. The high occurrence of halitosis in this study sample when compared with other studies that utilized methods other than the OralChroma^TM^ for VSCs measuring was not unexpected [5,6,7,8,19,31]. The capability of the OralChroma^TM^ to detect a low concentration of VSCs and its sensitivity to differentiate between gases of different origins justified this finding. Furthermore, children in this study were considered at a high caries risk and caries has been associated with halitosis [32]. 

Most of the parents who perceived halitosis in their children reported improvement following the FMR under GA, were halitosis disappeared or became less noticeable. Additionally, none of the parents reported any deterioration in oral breath from the group parents who did not perceive halitosis before the treatment. This finding is in agreement with El Batawai et al., who reported an overall improved oral health-related quality of life following FMR under GA, including significant reductions in the bad breath perceived by parents [24]. Amir et al. also reported that 63% of parents noted an improvement in bad breath after following oral hygiene instructions [17]. 

In the present study, a significant reduction in clinical halitosis (VSCs concentrations) was detected in more than half of the children following FMR under GA. Interestingly, similar findings were reported in the medical field, where halitosis in children has received considerable attention [29,33]. Choi et al. and Dinc et al. Used OralChroma^TM^ to evaluate the VSCs concentrations in children and adolescents before and after tonsillectomy and adenoidectomy, respectively, under GA. They reported that the concentrations of VSCs were decreased after the procedures [29,33]. The impact of FMR on the VSCs concentrations in this study could be related to the elimination of all dental cavities and caries by extracting grossly decayed teeth or remaining roots, restoring cavities with resin restorations or crowns, teeth cleaning and polishing, all of which lead to reduced anaerobic bacteria sources and food debris accumulation that together are responsible for VSCs formation [34]. The significant improvement noticed in the clinical parameters in this study may have impacted the results. This improvement is in agreement with previous studies reporting halitosis changes in children after an intervention or controlling a disease and altering a behavior [8,16,17,18]. Amir et al. reported a reduction in halitosis in children after providing oral hygiene instructions [17]. Additionally, Nalçaci and Sönmez reported lower halitosis levels upon improving oral hygiene and controlling gingival diseases in children [8]. Others have reported halitosis reductions in children after providing nonsurgical periodontal treatment and improving oral hygiene and tongue cleaning [16,18]. 

In the present study, two children developed clinical halitosis after rehabilitation, although none of their parents noticed halitosis after treatment. Those children showed severe gingival inflammation and poor oral hygiene after the FMR in the follow-up visit. 

Overall, the results of this study are consistent with the literature on the impact of FMR under GA on children. It is well documented that FMR under GA for children in a single session is an efficient and convenient method of treatment, where extensive procedures are delivered with minimal discomfort to the child, resulting in less long-term stress on both the child and family [21,22,35]. It also showed a positive impact on the OHRQoL in children in different aspects. Added to that, providing treatment under GA has become more acceptable by parents [36]. The effect of FMR on halitosis noted in this study was assessed only in the short term; further studies confirming this finding with long term follow-up are advised. Additionally, objective assessment of VSCs concentrations using OralChroma^TM^ in the OHRQoL studies is recommended. 

## 5. Conclusions

Based on the results of this study, the majority of parents (80%) reported improvement of perceived halitosis in their children following FMR under GA. Adding to that, FMR under GA showed significant reduction in VSCs concentrations below the level of detectable halitosis in more than half of children (56.3%). Also, significant improvement was noted in the following parameters after FMR under GA: MGI, WTCI and DI-S.

## Figures and Tables

**Table 1:** Descriptive of demographic data, parent’s perception and clinical halitosis before FMR.

Variables	Mean ± SD	Frequency (%)
Age	5.4 ± 1.6	
Child gender Male Female		
		18 (31.6%)
		39 (68.4%)
Questionnaire filled by Father Mother		
		41(72%)
		16(28%)
Parents perception of halitosis in their child Halitosis No Halitosis		
		34 (59.6%)
		23 (40.4%)
Clinical halitosis (OralChroma^TM^) Halitosis No Halitosis		
		48 (84.2%)
		9 (15.8%)

**Table 2:** Clinical halitosis before and after full mouth rehabilitation (FMR).

Clinical Halitosis		After FMR			*p*-Value
		No Halitosis	Halitosis	Total	
**Before FMR**	No Halitosis	7 (77.8)	2 (22.2)	9 (15.8)	<0.001 *
	Halitosis	27 (56.3)	21 (43.7)	48 (84.2)	
	Total	34 (59.6)	23 (40.4)	57 (100)	

* *p*-value for McNemar test.

**Table 3:** **VSCs** levels before and after FMR.

VSCs		Median (IQR)	*p*-Value *
H_2_S	Before FMR	225 (396)	<0.001
	After FMR	28 (141)	
CH_3_SH	Before FMR	62 (116)	<0.001
	After FMR	2 (24)	
CH_3_SCH_3_	Before FMR	10 (34)	<0.001
	After FMR	0 (3)	

* *p*-value for Wilcoxon singed ranks test.

**Table 4:** Clinical parameters before and after FMR.

Clinical Parameters	Before FMR	After FMR	*p*-Value
MGI N(%)			
• No gingival inflammation	34 (59.6)	48 (84.2)	0.001 **
• Inflamed gingiva	23 (40.4)	9 (15.8)	
DI median (IQR)	4 (1)	2 (1)	<0.001 *
WTCI median (IQR)	4 (1.5)	2 (2)	<0.001 *
Decayed median (IQR)	10 (4)	0	<0.001 *
Missing median (IQR)	0	4 (4)	<0.001 *
Filled median (IQR)	0	7 (4.5)	<0.001 *

* *p*-value for Wilcoxson signed ranked test; ** *p*-value for McNemar test.

## Data Availability

The data presented in this study are available on request from the corresponding author. The data are not publicly available.
